# Genetic variability, combining ability and molecular diversity-based parental line selection for heterosis breeding in field corn (*Zea mays* L.)

**DOI:** 10.1007/s11033-022-07295-3

**Published:** 2022-04-26

**Authors:** Ganapati Mukri, Meghashri S Patil, Babu N Motagi, Jayant S Bhat, Chandu Singh, S. P. Jeevan Kumar, R. N Gadag, Navin C Gupta, Jesus Simal-Gandara

**Affiliations:** 1 Division of Genetics, ICAR-Indian Agricultural Research Institute, 110012 New Delhi, India; 2 Department of Genetics and Plant Breeding, University of Agricultural Science, 580005 Dharwad, India; 3 ICAR-Directorate of Floricultural Research, 411036 Pune, Maharashtra India; 4 ICAR-National Institute of Plant Biotechnology, 110012 New Delhi, India; 5 Nutrition and Bromatology Group, Department of Analytical Chemistry and Food Science, Faculty of Science, Universidade de Vigo, E32004 Ourense, Spain

**Keywords:** Corn hybrid, Combining ability, Heterosis, Molecular diversity, Parental line selection

## Abstract

**Background:**

The demand of maize crop is increasing day by day, hence to reduce the production and demand gap, there is a need to extract the high yielding parental lines to improve *per se* yield of the hybrids, which could help to enhance the productivity in maize crops.

**Methods and results:**

The present investigation was carried out to select the best medium maturing inbred lines, among a set of 118 inbred lines. Based on the Duncan multiple range test, out of 118 lines, 16 inbred lines were selected on the basis of its high yield *per se* and flowering time. The molecular diversity was carried out using SSR markers linked to heterotic QTL and up on diversity analysis it classified selected genotypes in to three distinct groups. Among the selected inbred lines, a wider genetic variability and molecular diversity were observed. A total of 39 test crosses were generated after classifying 16 inbred lines in to three testers and thirteen lines (based on *per se* grain yield and molecular diversity) and crossing them in line × tester manner.

**Conclusion:**

Combining ability analysis of these parental lines showed that female parents, PML 109, PML 110, PML 111, PML 114 and PML 116 showed additive effect for KRN and grain yield, whereas male parents, PML 46, and PML 93 showed epistatic effect for KRN and PML 102 showed epistatic effect for grain yield. The generated information in the present investigation may be exploited for heterosis breeding in filed corn.

**Key messages:**

To tackle the balanced dietary requirement of Indian population; we focused to enhance the productivity of maize hybrids using genetically broad based, elite, diverse inbred lines. Combination of selection criterion, not only augment the productivity but also improves the quality of hybrid/s.

**Supplementary Information:**

The online version contains supplementary material available at 10.1007/s11033-022-07295-3.

## Introduction

Maize is one of the key cereals, which plays the major role in Indian agriculture, especially to meet the staple food, livestock feed, edible oil and biofuel demand of growing population and industry. Hybrids play crucial role in maize productivity, which not only enhance production but also alleviate food scarcity and nutrition requirement of the developing countries. Hybrid maize cultivars development needs selection of appropriate parents (inbred lines) which is the concealed of success in hybrid maize development. Identification of high yielding hybrids require careful selection of parents based on their combining ability and underlying genetic constituents of inbred lines [1].

The extent of enhancement of maize productivity not only depend on the genetic variability but also diversity of the parental inbred lines involved in the cross combination, which ultimately determine the magnitude of heterosis. The power of heterosis by breeding filial one (F_1_) hybrids exhibiting superior vigor for plant growth and grain yield was first exploited in maize. Though the mystery of heterosis has been explored for over a century, but the underlying mechanism remains insufficiently understood [2]. For the better exploitation of heterosis, systematic selection of parental lines followed by the identification of superior hybrid combinations plays crucial role. However, the extent of heterosis varies with the genetic distance of parents, mode of reproduction, nature of traits under investigation and prevailing environment in which parental lines perform well [3].

Execution of specific methodology is very important to identify suitable parental lines for hybrid breeding as different genetic approaches are available to identify diverse parents or to determine genetic distance among the genotypes. With the advent of molecular markers, identification of genetic diversity followed by establishment of genetic relation has made easy to select the parents with exploitable genetic diversity. However, breeder should depend not only on the genetic diversity of the parents, as in different crops, contradictory results have been reported with respect to the relationship between genetic distance and heterosis [3]. This might be due to the fact that apart from the genetic diversity, heterosis is also dependent on the relevant considerations of direction and magnitude of dominance, biological feasibility and the type of gene action exhibited by the inbred lines involved in the hybrid combinations. On the other hand, the combining ability analyses for traits under investigation were also equally important to capture other variance, which explains the extent of heterosis and makes the parental selection much more effective. Hence, it was realized that the measures of both general combining ability (GCA) and specific combining ability (SCA) are necessary for the selection of parental lines to develop heterotic combinations [4].

In recent years researchers have used quantitative genetics, physiology, and molecular approaches in an effort to understand the basis of heterosis [5]. But the explanation of the concept of heterosis is meaning less without understanding the genetic composition of parental lines used in the development of hybrids [6]. From the previous experiences it was very clear that any single criterion adapted for the selection of inbred line would not yield potential inbred lines with high exploitable heterosis among them [7].

Therefore, the present investigation was emphasized to explore potential inbred parental lines based on holistic approach with genetic variability, combining ability and molecular diversity studies which could be help to frame the heterosis breeding program in field corn.

## Materials and methods

### Phenotypic selection of parental lines

A set of 118 stabilized maize inbred lines, which were derived from the diverse source of population and evaluated with two rows each of genotypes along with reiterating high yielding checks at regular interval using augmented block design during 2016 and 2016–17 at ICAR-Regional Research Centre, Dharwad, Karnataka. These inbred lines were categorized into different maturity groups based on their flowering time. The Duncan multiple range test was used for selection, out of 118 inbred line, 16 top performing medium maturing inbred lines were selected based on yield *per se* and maturity (Supplementary Table 1).

The selected 16 inbred lines were evaluated in replicated trials under Randomised Complete Block Design (RCBD) with two rows each of test genotypes at ICAR-Indian Agricultural Research Institute, New Delhi during 2017 and used for further investigation. The recommended package of practices was followed to raise a healthy crop. Data on grain yield component traits viz., cob length (CL) (cm), cob girth (CG) (mm), kernel row number (KRN) and kernel per row (KPR) were recorded along with grain yield (kg/ha) using standard methodology and data were analysed through SAS 9.3v software (http://stat.iasri.res.in/sscnarsportal).

## Heterotic QTL based molecular marker diversity in the parental lines

Heterotic QTL based markers with high LOD value (˃ 4) were selected and primer details were collected from Maize GDB (www.maizegdb.org). A total of 50 linked SSR markers (Supplementary Table 2) distributed across the different chromosomes (1–10) were used for molecular diversity analysis. The genomic DNA of 16 parental lines was isolated using CTAB (Cetyl-trimethyl ammonium bromide) method [8]. The PCR was performed with 1 unit of *Taq* DNA polymerase (GeneDireX, Inc.), 10X reaction buffer (GeneDireX, Inc.), 0.1 mM dNTPs, 10 pmol/ µL each primer and 50 ng DNA template in a total reaction volume of 25 µL. The PCR amplification was carried out with initial denaturation at 94 ^°^C for 5 min. followed by 35 cycles consisting of denaturation at 94 ^°^C for 30s, annealing at 55 ^°^C for 30s, extension at 72 ^°^C for 60s and a final extension of 7 min. at 72 ^°^C. The PCR amplified fragments were resolved o 3.5% (w/v) agarose gel (HiMedia) and the amplified products were scored the estimated polymorphism information content (PIC) values as per Anderson et al. 1993 [9]. The molecular data was subjected for diversity analysis using DARwin software [10].

## Development of hybrids and their evaluation

The inbred lines selected in the field experiment were classified as female (13) and male (three) parental lines based on their variability and molecular diversity. A set of 39 test cross hybrids were generated using these parental lines by following line × tester mating design at ICAR-Indian Agricultural Research Institute, New Delhi, during 2017–18. The generated test cross hybrids were evaluated in RCBD with two replications at Regional Agricultural Research Station, Vijayapura (16^°^ 49’ N latitude, 75^°^ 43’ E longitude and 593 mean sea level) during 2018.

These hybrids were raised in paired rows of three-meter length with a spacing of 60 × 20 cm. The standard agronomical package of practices was followed to raise the healthy crops. The data was recorded using five randomly selected plants (from each entry/replication), competitive plants were tagged and numbered in the middle row to observe yield and other quantitative characters. Data were recorded on CL (cm), CG (cm), KRN, KPR and grain yield (kg/ha) at respective stages of growth and development of the crop. The software, TNAUSTAT was used to estimate the combing ability and other descriptive statistics [11].

## Results

### Genetic variability of parental lines

Analysis of variance of 16 inbred lines indicated wider variability and significant differences among each other for the trait under consideration (Table 1). The mean cob length recorded was 12.83 cm which ranged from 8.75 to 16.05 cm. For cob girth average value was 35.47 mm and range was 26.25 to 41.35 mm. The KRN and KPR are the other related, complimenting and important yield attributing traits. For these characters (KRN and KPR) recorded mean value of 14 and 22, respectively with the range of 10–22 and 12–31, respectively. The mean *per se* grain yield recorded was 2365 kg/ha which ranged from 1087 to 3113 kg/ha (Supplementary Table-3).

**Table 1:** Analysis of variance for yield component traits of parental lines

Sources of variation	MSS				
	DF	Cob Girth	Cob Length	Kernel Per Row	Kernel Row Number
Replication	1	0.19	0.43	0.47	0.50
Genotypes	15	47.62**	9.10**	50.28**	12.23**
Error	15	0.18	0.05	2.44	1.03

DF: Degree of freedom, MSS: Mean sum of squares

## Molecular diversity of parental lines

**Fig. 1:**
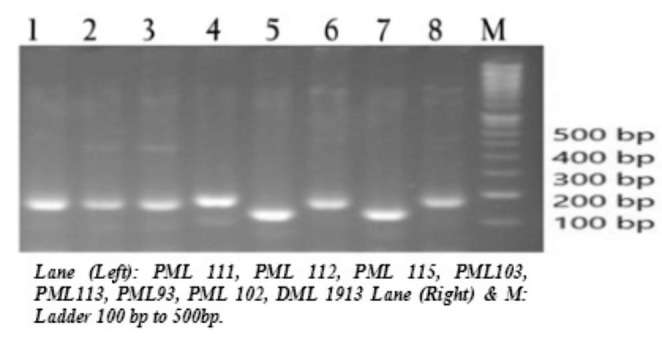
Gel image showing polymorphic survey among inbred lines

The molecular diversity analysis was carried out using simple sequence repeat (SSR) markers, which is linked to yield and heterotic QTLs in maize. These linked markers (50 no.s) were used for polymorphic survey using a set of 16 inbred lines to understand the molecular diversity among the lines. These markers found highly polymorphic among parental lines (Fig. 1). The polymorphic information content (PIC) value of these markers was > 0.5 with range of value 0.62–0.99 (Supplementary Table 4).

The cluster analysis using molecular profile generated by SSR markers, classified inbred lines into three distinct clusters (Supplementary Fig. 1). Cluster I had eight inbred lines (PML 44, PML 93, PML 103, PML 111, PML 112, PML 115, DML 1913 and DML 1336), cluster II had seven inbred lines (PML 45, PML 102, PML 109, PML 110, PML 113, PML 114 and PML 116) and the cluster III was mono-genotypic (PML 46). The clusters mean for the grain yield was 2196.40 kg/ha, 2464.06 kg/ha and 3026.25 kg/ha, for Cluster I, Cluster II and Cluster III respectively. Similarly, cluster mean for CL (13.55, 11.45, 16.05 cm), for CG (34.92, 36.57, 31.50 mm) and for KRN (22.01, 19.36 and 30.38), respectively were also recorded by these clusters (Supplementary Table 5). This indicated that the inbred lines belonging to these clusters have substantial genetic diversity.

## Genetic variability of hybrids

The analysis of variance for morpho-physiological and yield related traits among 39 test hybrids obtained by the crossing of 13 inbred lines with three testers, showed that the mean sum of squares due to the traits studied were highly significant, indicating the presence of substantial differences among the hybrids for all the studied traits (Table 2). Variance due to testers and crosses were highly significant differences.

**Table 2:** Analysis of variance for line x tester for yield and yield component traits

Source	MSS					
	Df	Cob length (cm)	Cob girth (cm)	Kernel per row	Kernel row number	Grain yield (t/ha)
Replication	2	7.05	1.79	37.85	1.89	0.23
Genotypes	43	3.15*	0.78*	16.55*	2.60**	1.11**
Cross	38	3.26*	0.83*	17.48*	2.78**	0.99**
Line	12	1.90*	1.12*	12.47	3.77**	1.32**
Tester	2	11.49*	2.36*	4.88	6.37*	1.72**
LXT	24	3.24	0.56	21.04	1.99	0.76**
Error	86	3.10	0.76	12.76	1.54	0.26
CV (%)		10.19	5.95	11.20	9.06	16.70
CD at 5%		2.82	1.39	5.79	2.07	0.813
CD at 1%		3.75	1.85	7.69	2.75	1.08

Df: degrees of freedom, * & **: Significance at 5% and 1% probability, respectively

## Combining ability of parental lines

General combining ability indicates the average performance of the lines in a series of cross combination. Among the tested lines, the PML 116 is having the significant positive GCA effect for cob girth (0.61) followed by PML 110 (0.59), PML 111 (0.57), PML 114 (0.53) and PML 109 (0.46). For the KRN, the lines PML 116 is having the significant positive GCA effect (1.28) followed by PML 110 (1.15), PML 111 (1.01), PML 109 (0.85) and PML 114 (0.82). Hence, these are good general combiners for cob girth and KRN traits. Similarly, PML 109, PML 110, PML 111, PML 114, and PML 116 with significant GCA effects of 0.38, 0.46, 0.41, 0.36 and 0.56 respectively, found good general combiner for grain yield.

Among the testers used, PML 46 and PML 93 had non-significant GCA effect for KRN (-0.33 and − 0.12) and grain yield (0.12 and 0.12). However, PML 102, other inbred line used as tester, showed significant GCA effect for both KRN (0.45) and grain yield (-0.24) (Table 3). For KRN, the hybrids AH-4316 (PML109×PML93), AH-4304 (PML110×PML46), AH-4305 (PML111×PML46), AH-4334 (PML114×PML102) and AH-4323 (PML116×PML93) were recorded significant SCA effects in positive direction. In case of grain yield, the hybrid AH-4323 (PML116×PML93) is having the significant SCA effect followed by AH-4316 (PML109×PML93), AH-4304 (PML110×PML46), AH-4305 (PML111×PML46) and AH-4334 (PML114×PML102) in positive direction (Table 4).

**Table 3:** The GCA effects of yield related traits and per se yield of parental lines

Parents	Cob length (cm)	Cob girth (cm)	Kernel row number	Kernel Per row	Grain yield (t/ha)	Grain Yield (kg/ha)
**Lines**						
PML 45	-0.05	-0.36	-0.86 *	-0.06	-0.38 *	2698.55
PML 44	0.07	-0.29	-0.68	-0.64	-0.48 **	2207.00
PML 103	0.07	0.40	0.43	0.14	-0.49 **	2715.00
PML 109	0.16	0.46*	0.85*	0.23	0.38*	2447.00
PML 110	0.32	0.59*	1.15**	0.85	0.46**	2741.35
PML 111	0.11	0.57*	1.01**	0.36	0.41 *	2730.10
PML 112	-0.21	0.21	0.28	-0.46	0.31	2604.50
PML 113	-0.22	-0.36	0.25	0.85	-0.03	2433.50
PML 114	0.18	0.53*	0.82*	0.17	0.36 **	2178.93
PML 115	-0.03	-0.17	-0.68	0.41	0.26	1722.39
PML 116	0.42	0.61*	1.28**	0.59	0.56**	1713.86
DML-1336	0.25	-0.14	0.39	1.07	0.01	1087.99
DML-1913	-0.63	-0.30	-0.32	-3.28 **	-0.62 **	1390.95
SE	0.59	0.29	0.43	1.17	0.18	-
**Testers**						
PML 46	0.51	0.01	-0.33	-0.01	0.12	3113.20
PML 93	-0.57 *	0.25	-0.12	-0.35	0.12	3035.25
PML 102	0.07	-0.24	0.45 *	0.36	-0.24 **	3026.25
SE	0.29	0.14	0.21	0.56	0.08	-

SE: Standard error, * and **: Significance at 5% and 1% probability, respectively

**Table 4:** The *SCA* effects of hybrids for yield and yield component traits

S.No	Hybrids	Pedigree	Cob length (cm)	Cob girth (cm)	Kernel row number	Kernel Per row	Grain yield (t/ha)
1	AH4300	PML 45XPML 46	-0.26	-0.34	-0.52	0.56	-0.06
2	AH4313	PML 44XPML 46	-1.27	-0.40	-0.19	-2.16	-0.07
3	AH4326	PML 103XPML 46	1.53	0.74	0.70	1.60	0.13
4	AH4301	PML 109XPML 46	0.62	0.10	0.64	1.27	-0.03
5	AH4314	PML 110XPML 46	-1.34	-0.42	-0.50	-2.98	-0.10
6	AH4327	PML 111XPML 46	0.72	0.32	-0.14	1.71	0.13
7	AH4302	PML 112XPML 46	-0.15	-0.06	-0.07	-0.37	-0.58
8	AH4315	PML 113XPML 46	-0.50	0.35	0.79	0.71	0.58
9	AH4328	PML 114XPML 46	0.65	-0.29	-0.72	-0.34	0.01
10	AH4303	PML 115XPML 46	-0.39	0.17	0.51	1.03	-0.18
11	AH4316	PML 116XPML 46	1.34	1.04	1.17*	2.17	0.68*
12	AH4329	DML-1336XPML 46	-0.95	-0.03	0.26	-3.20	-0.29
13	AH4304	DML-1913XPML 46	1.25	1.17	1.31*	2.85	0.85 **
14	AH4317	PML 45XPML 93	0.14	-0.21	-0.50	-2.27	-0.02
15	AH4330	PML 44XPML 93	-0.39	-0.56	-0.41	-0.58	-0.82 **
16	AH4305	PML 103XPML 93	1.24	1.12	1.28*	2.76	0.81**
17	AH4318	PML 109XPML 93	-0.39	-0.17	-0.32	0.82	-0.36
18	AH4331	PML 110XPML 93	0.73	0.37	0.44	-0.16	-0.02
19	AH4306	PML 111XPML 93	0.49	0.46	-0.12	-1.44	-0.18
20	AH4319	PML 112XPML 93	-0.43	-0.28	1.01	-0.29	0.04
21	AH4332	PML 113XPML 93	-0.07	-0.18	-0.90	1.73	0.14
22	AH4307	PML 114XPML 93	0.23	-0.04	0.11	0.38	0.16
23	AH4320	PML 115XPML 93	0.99	0.38	0.83	0.66	-0.51
24	AH4333	PML 116XPML 93	-1.22	-0.34	-0.94	-1.05	0.35
25	AH4308	DML-1336XPML 93	-0.89	-0.50	-0.83	-2.64	0.00
26	AH4321	DML-1913XPML 93	0.74	0.29	-0.23	3.97	-0.44
27	AH4334	PML 45XPML 102	1.15	1.00	1.06*	1.94	0.53*
28	AH4309	PML 44XPML 102	-0.75	-0.56	-0.03	-3.84	0.30
29	AH4322	PML 103XPML 102	0.66	0.20	-0.37	0.31	-0.34
30	AH4335	PML 109XPML 102	0.09	0.36	0.39	3.53	0.03
31	AH4310	PML 110XPML 102	-0.96	-0.24	-1.14	-0.90	-0.17
32	AH4323	PML 111XPML 102	1.29	1.24	1.48*	2.96	0.99 **
33	AH4336	PML 112XPML 102	1.25	-0.01	1.42	2.86	-0.81 **
34	AH4311	PML 113XPML 102	0.47	0.38	0.37	2.43	-0.01
35	AH4324	PML 114XPML 102	0.25	0.07	0.70	-2.23	-0.28
36	AH4337	PML 115XPML 102	-0.72	-0.45	-1.07	-0.20	0.29
37	AH4312	PML 116XPML 102	1.68	0.04	0.28	1.32	-0.47
38	AH4325	DML-1336XPML 102	0.09	0.09	-0.19	3.26	0.04
39	AH4338	DML-1913XPML 102	-1.78	-0.13	-0.10	-4.58 *	0.44
SE			1.03	0.50	0.75	2.03	0.30

SE: Standard error, * and **: Significance at 5% and 1% probability respectively

Comparative evaluation of promising combinations having high SCA for grain yield was carried out. The five hybrid combinations viz., AH-4323 (PML 116 × PML 93), AH-4316 (PML 110 × PML 46), AH-4304 (PML 111 × PML 46), AH-4305 (PML 109 × PML 93) and AH-4334 (PML 114 × PML 102) showed significantly superior grain yield over the medium maturing national check hybrid, Bio-9544 (Supplementary Table 6).

## Discussion

Breeding for hybrid in any crop is one of the finest interventions of agriculture innovation which has directly impact on increasing in productivity. Understanding heterosis from the perspective of any single mechanisms alone may be elusive, because heterosis is likely an emergent property of populations [7]. Hybrid breeding technology mainly involves development of stable, trait specific inbred parental lines and identification of suitable parent for heterosis breeding [4]. Genetic variability is the pre-requisite for the selection of inbred lines that leads to the directed maize improvement [12]. In the present study, 16 promising inbred lines were selected among the 118-field corn inbred lines, evaluated across two seasons. The analysis of variance indicated the presence of high genetic variability for CL, CG, KRN, KPR and grain yield. Grain yield being the function of yield component traits selected majorly for the enhancement of productivity. Hence, for the first instance, lines viz., PML 46, PML 93 and PML 102 with their grain yield, 3113.20, 3035.25, 3026.25 kg/ha respectively were selected as high yielding inbred lines, considering the population mean for the grain yield (2365.36 kg/ha) and its standard deviation (605.52 kg/ha) (Supplementary Table 3).

Heterosis is the function of allelic diversity and degree of dominance of a trait harbor in the parental lines, which are exploited during the development of hybrids [13]. Allelic diversity that explained by molecular diversity along with the morphological parameters gives better insights to understand the genetic base of the inbred lines under selection. Molecular diversity analysis (50 SSR markers) showed PIC value > 0.5, which indicated that all sixteen inbred lines were highly diverse among each other [14]. Further, the cluster analysis was done using same markers (linked to yield and heterotic QTLs), it was showed three distinct clusters (I, II, & III), which showed the wider genetic divergence among the inbred lines under study.

The potentiality of inbred lines favoring heterosis can be identified by their combining ability studies. In the present investigation, a set of 13 female (lines) and 3 male (tester) parental lines were identified and crossed into line x testers fashion and generated 39 test cross hybrids. The analysis of variance for combining ability suggested that there was significant variation due to cross or entries for all the traits studied, which in turn suggested the presence of wider genetic diversity among different traits. Furthermore, the partitioning of the mean sum of squares attribute to different sources of variation revealed that mean sum of squares due to lines and its crosses were highly significant. Also, there was significant variation due to lines and testers for all the traits under studied except KPR; hence, there is a high genetic divergence between lines and testers [13]. This indicted that contribution of lines and testers for the final grain yield may be traits other than through number of KPR.

The interaction between line and tester was showed significant differences for grain yield trait than the rest of traits. Therefore, testers used in the hybrid combinations were better differentiated for productivity, the contribution towards variance due to hybrids could be better accounted for grain yield. Hence, as advocated, this design gives better insights to the performance of the lines and testers involved in the series of cross combinations [15].

The complete understanding of genetic basis of heterosis and combining ability remains elusive, which, however, does not affect the vital role of heterosis and combining ability in general, and maize breeding, in particular. Although there are still some gaps to understand the mechanism of heterosis, but great progress has been made in predicting hybrid performance based on the combining ability studies [16]. In the present study, similar effort was made to understand the lines performance through their combing ability studies, which may be helpful in future breeding program and/or selecting parental lines to exploit maximum heterosis [17].

Among the testers, PML 102 shows significant positive GCA effect for KRN (0.45) and negative GCA effect for yield (-0.24). Similarly, PML 93 and PML 46 showed non-significant positive GCA effect for KRN and yield [18]. Therefore, above-mentioned testers can be used for the better utilization of these yield components through the strategy of heterosis breeding. Also, genotypes with high GCA effect with desirable traits can be used to constitute a good source population to derive better inbred lines and/or as a donor (KRN and CG) for further improvement of inbred lines [19]. As enunciated, GCA is an effective tool in the selection of parents based on the performance of their progenies [20]. A low GCA value, positive or negative, implies that the mean of a parent in crossing with the other does not vary largely from the general mean of the crosses [21]. In contrast, a high GCA value implies that parental mean is either superior or inferior to the general mean in cross combinations. This is a potent evidence of desirable gene flow from parents to offspring at high intensity and represents information regarding the concentration of predominantly additive genes [22]. The combining ability analysis is one of the best methods for evaluating parental performance and understanding the dynamics of genes involved in trait expression and has been successfully utilized in crop breeding [23]. Parental GCA estimates in desirable direction also indicative of their potentiality in generating promising breeding populations.

The usefulness of a particular cross involving diverse parental lines in exploiting heterosis phenomenon is judged by the SCA effect of the component lines. According to Sprague and Tatum [24], SCA is controlled by non-additive gene action and it can be utilized to determine specific heterotic crosses for the respective trait of interest [25]. Hence, the SCA effect is an important criterion for the evaluation of hybrids to select trait specific cross combinations [23]. In the present study, it was found that the hybrids, AH-4316, AH-4304, AH-4305, AH-4334 and AH-4323 were having significant SCA effects in positive direction for traits for KRN and grain yield in desirable direction. Further, it was observed that, female parents had positive and significant GCA effect for KRN and grain yield. The significantly high SCA observed for the test cross may also be attributed to good combiner parent, depicting its favorable additive effects, but the poor combiner parental genotype displaying the epistatic effects [22]. These results clearly indicated that breeder pertinent to maize improvement must pay attention of SCA and GCA components for selection of elite inbred parental lines for the development of heterotic hybrids. Hence, it was also observed that complementary gene complexes may involved in expression of heterosis among the parental lines [25].

Although *per se* performance of female inbred lines plays crucial role in economic seed production of any hybrids, breeder may tend to select even poor performing female parents, if genetic distance between the parents is high, will be explore for heterosis breeding programme [26, 27]. The extent of heterosis has been reported to vary with genetics of traits under consideration [28–32]. In the study, with the comparatively poor *per se* grain yield, the female inbred line PML 116 had contributed promising hybrids, as this line was having significant GCA with additive effect for KRN and grain yield. Hence, breeder should trade-off between *per se* performance of the lines and their genetic diversity after repeated evaluation.

## Conclusions

Understanding the breeding value of the parental lines in hybrid breeding program plays a paramount role in increasing hybrid yield *per se*. Test cross performance gives some idea about breeding value of the lines under testing. The present investigation identified the wider genetic variability among inbred lines under study. Based on combining ability analysis (GCA and SCA), inbred line showed both additive and epistatic effect for yield and yield attribute traits and also showed distinct divergence in inbred lines based on molecular diversity approach.

## Electronic Supplementary Material

Below is the link to the electronic supplementary material.

Supplementary Material 1

## Data Availability

The data are available with the corresponding author and upon request they will be provided.
