# Supplementary material for: Genetic variability, combining ability and molecular diversity-based parental line selection for heterosis breeding in field corn (Zea mays L.)
Source: Mol Biol Rep. 2022 Apr 26;49(6):4517–24. doi: 10.1007/s11033-022-07295-3 (PMC9262758; doi:10.1007/s11033-022-07295-3)
Supplement: Supplementary file 1 — Supplementary Material 1 [file 11033_2022_7295_MOESM1_ESM.docx]

Supplementary Table 1. Detailed information on the 118 inbred lines used in the initial screening for high yield *per se*

| S No. | Nomenc  lature | Pedigree | S. No. | Nomenc  lature | Pedigree | S.No. | Nomenc  lature | Pedigree | S.No | Nomenc  lature | Pedigree |
| --- | --- | --- | --- | --- | --- | --- | --- | --- | --- | --- | --- |
| 1 | PML 1 | Geo.Pre. Dia.-2-2-4 | 31 | PML 31 | PAC-745-9-2-2 | 61 | PML 61 | (HKI1105 X CML170)-1-2 | 91 | PML 91 | V373-V6-17 |
| 2 | PML 2 | Geo.Pre. Dia.-12-1-2 | 32 | PML 32 | PAC-745-9-2-4 | 62 | PML 62 | (CE8 X CE16)-2 | 92 | PML 92 | PAC-753-13-1-3 |
| 3 | PML 3 | KMH-25K60-2-1-2 | 33 | PML 33 | PAC-745-9-3-4 | 63 | PML 63 | (KML27 X V335)-3-1-1 | 93 | PML 93 | KDMH-176-5-1-1 |
| 4 | PML 4 | KMH-25K60-2-1-8 | 34 | PML 34 | PAC-745-12-1-1 | 64 | PML 64 | (KML27 X V335)-2-1-1 | 94 | PML 94 | PAC-740-10-1-1 |
| 5 | PML 5 | KMH-25K60-12-1-2 | 35 | PML 35 | PAC-745-12-1-2 | 65 | PML 65 | (CE13 X HKI1128)-2-1-2 | 95 | PML 95 | P-3501-10-1-3 |
| 6 | PML 6 | KH-2192-10-1-1 | 36 | PML 36 | PAC-745-15-1-1 | 66 | PML 66 | IML307-1-1-1 | 96 | PML 96 | V931-16 |
| 7 | PML 7 | KH-2192-14-1-2 | 37 | PML 37 | PAC-753-4-1-1 | 67 | PML 67 | IML307-2-1-1 | 97 | PML 97 | V931-16 |
| 8 | PML 8 | GEO-2101-15-1-4 | 38 | PML 38 | PAC-753-7-1-3 | 68 | PML 68 | RMH-932-3-1-1 | 98 | PML 98 | V929-7 |
| 9 | PML 9 | POLO-1-2-2 | 39 | PML 39 | PAC-753-8-2-2 | 69 | PML 69 | SFAL-X2-11-1-5 | 99 | PML 99 | V929-7 |
| 10 | PML 10 | POLO-1-2-3 | 40 | PML 40 | PAC-753-9-1-6 | 70 | PML 70 | GEO-2101-1-1-1 | 100 | PML 100 | Z485-22 |
| 11 | PML 11 | POLO-14-1-2 | 41 | PML 41 | PAC-753-9-1-7 | 71 | PML 71 | GEO-2101-15-1-4 | 101 | PML 101 | SN-194-29 |
| 12 | PML 12 | POLO-14-1-3 | 42 | PML 42 | PAC-753-12-1-1 | 72 | PML 72 | KDMH-4086-15-1-5 | 102 | PML 102 | KDMH-755-12-2-1-1 |
| 13 | PML 13 | KDMH-4086-15-1-5 | 43 | PML 43 | PAC-753-12-1-4 | 73 | PML 73 | KDMH-4086-15-1-7 | 103 | PML 103 | CP-888-14-2-2-2 |
| 14 | PML 14 | KDMH-4086-15-1-7 | 44 | PML 44 | PAC-753-13-1-2 | 74 | PML 74 | RASI-3033-15-1-1 | 104 | PML 104 | PAC-753-13-6-3 |
| 15 | PML 15 | CMH-08-282-14-1-1 | 45 | PML 45 | PAC-753-13-1-4 | 75 | PML 75 | PAC-753-8-1-3 | 105 | PML 105 | LG-3281-11-2-1 |
| 16 | PML 16 | HQPM5-13-1-1 | 46 | PML 46 | SAFAL-X12-9-1-1 | 76 | PML 76 | PAC-753-9-1-7 | 106 | PML 106 | SFAL-X2-11-3-1-5 |
| 17 | PML 17 | RMH-3591-1-1-1 | 47 | PML 47 | 30B07-9-4 | 77 | PML 77 | SUN-234-5-2-1 | 107 | PML 107 | GEO-2101-15-2-1-4 |
| 18 | PML 18 | RMH-3591-4-1-1 | 48 | PML 48 | 115-08-01-6-1 | 78 | PML 78 | P-3501-3-1 | 108 | PML 108 | POLO-11-2-1-2-1 |
| 19 | PML 19 | RMH-3591-4-1-2 | 49 | PML 49 | P-3501-3-1 | 79 | PML 79 | LG-3281-11-1-1 | 109 | PML 109 | PAC-753-9-1-3-2-1 |
| 20 | PML 20 | RMH-3591-4-1-3 | 50 | PML 50 | P-3501-5-2 | 80 | PML 80 | (BML6 X CE18)-1-7 | 110 | PML 110 | POLO-14-1-2 |
| 21 | PML 21 | DMH-119-1-1-4 | 51 | PML 51 | PMH3-11-1 | 81 | PML 81 | (VQL-1 X V-373)-1-1-1 | 111 | PML 111 | HQPM5-13-3-1 |
| 22 | PML 22 | DMH-119-10-1-5 | 52 | PML 52 | EH 1974-6-3 | 82 | PML 82 | PAC-745-6-1-1 | 112 | PML 112 | RMH-3591-14-1-2 |
| 23 | PML 23 | CP-999-9-1-3 | 53 | PML 53 | KH-517GOLD-9-1-1 | 83 | PML 83 | PAC-753-9-1-1 | 113 | PML 113 | PAC-753-4-1-1 |
| 24 | PML 24 | CP-999-15-2-4 | 54 | PML 54 | KDMH-755-12-1-1 | 84 | PML 84 | PAC-753-13-1-2 | 114 | PML 114 | 115-08-01-10-2 |
| 25 | PML 25 | KMH-218PLUS-1-1-3 | 55 | PML 55 | KDMH-755-12-1-2 | 85 | PML 85 | IML307-3-1-1 | 115 | PML 115 | KRISHNA GOLD -8-1-1 |
| 26 | PML 26 | RASI-3033-2-1-1 | 56 | PML 56 | HM4-10-1-3 | 86 | PML 86 | CP-888-14-1-1 | 116 | PML 116 | RMH-3591-14-5-2 |
| 27 | PML 27 | RASI-3033-15-1-1 | 57 | PML 57 | NK-6240-6-1-4 | 87 | PML 87 | CP-888-14-1-2 | 117 | DML-1913 | WNC-25-3-3 |
| 28 | PML 28 | PAC-745-2-1-1 | 58 | PML 58 | 900M-GOLD-7-1-2 | 88 | PML 88 | POLO-11-2-1 | 118 | DML-1336 | JCY-36-2-2-1-1 |
| 29 | PML 29 | PAC-745-2-1-2 | 59 | PML 59 | (BML6 X CE18)-1-2 | 89 | PML 89 | PAC-745-6-1-1 |  | - | - |
| 30 | PML 30 | PAC-745-9-2-1 | 60 | PML 60 | (BML6 X CE18)-1-3 | 90 | PML 90 | PAC-745-6-2-1 |  | - | - |

Supplementary Table 2. Detailed information on choice of SSRs and also the motif  [31-33]

| Marker | Traits | locus | Repeats | Forward primer | Reverse primer | References |
| --- | --- | --- | --- | --- | --- | --- |
| bnlg1006 | yield and heterosis | 5 | AG(20) | [GACCAGCGTGTTGATCCC](https://www.maizegdb.org/data_center/primer?id=171030) | [GGAGACCCCGACTCTCTCTC](https://www.maizegdb.org/data_center/primer?id=171031) | Fernandes et al. 2015 |
| bnlg1045 | yield and heterosis | 2.07 | AG(23) | [TCCCCGATAGCATATCGATC](https://www.maizegdb.org/data_center/primer?id=171068) | [GTGACTTTGGGGAGTTTGGA](https://www.maizegdb.org/data_center/primer?id=171069) | Fernandes et al. 2015 |
| Bnlg1064 | yield/yield heterosis | 2.03 | AG(16) | [CTGGTCCGAGATGATGGC](https://www.maizegdb.org/data_center/primer?id=171082) | [TCCATTTCTGCATCTGCAAC](https://www.maizegdb.org/data_center/primer?id=171083) | Xu, S.-X, Liu, J. and Liu, G.S. 2004 |
| Bnlg1074 | yield/yield heterosis | 10.04 | AG(14) | [CATGCTAATAGCCTACCGGG](https://www.maizegdb.org/data_center/primer?id=171092) | [TTTCCCCCTGATTCGTTATG](https://www.maizegdb.org/data_center/primer?id=171093) | Xu, S.-X, Liu, J. and Liu, G.S. 2004 |
| Bnlg1108 | yield/yield heterosis | 3.08 | AG(21) | [GGATTCCTTTATGACGGGGT](https://www.maizegdb.org/data_center/primer?id=171108) | [AGTAACAACCAAGGCATCGG](https://www.maizegdb.org/data_center/primer?id=171109) | Xu, S.-X, Liu, J. and Liu, G.S. 2004 |
| Bnlg1138 | yield/yield heterosis | 2.06 | AG(14) | [TGCTCTAGCCGACCTCAATT](https://www.maizegdb.org/data_center/primer?id=171130) | [ATGCCTGAACCGTGATTAGG](https://www.maizegdb.org/data_center/primer?id=171131) | Xu, S.-X, Liu, J. and Liu, G.S. 2004 |
| Bnlg1175 | yield/yield heterosis | 2.04 | AG(38) | [ACTTGCACGGTCTCGCTTAT](https://www.maizegdb.org/data_center/primer?id=171160) | [GCACTCCATCGCTATCTTCC](https://www.maizegdb.org/data_center/primer?id=171161) | Xu, S.-X, Liu, J. and Liu, G.S. 2004 |
| Bnlg1178 | yield/yield heterosis | 1.02 | AG(16) | [ACTACAGTTGAACGCCCCTG](https://www.maizegdb.org/data_center/primer?id=171164) | [GCTCATGTGCAAATGCAAGT](https://www.maizegdb.org/data_center/primer?id=171165) | Xu, S.-X, Liu, J. and Liu, G.S. 2004 |
| Bnlg1297 | yield/yield heterosis | 2.02 | AG(32) | [TCTCGATCGCTCCGATCTAT](https://www.maizegdb.org/data_center/primer?id=171230) | [GACTCAACTCCAAAAGGCGA](https://www.maizegdb.org/data_center/primer?id=171231) | Xu, S.-X, Liu, J. and Liu, G.S. 2004 |
| Bnlg1429 | yield/yield heterosis | 1.02 | AG(20) | [CTCCTCGCAAGGATCTTCAC](https://www.maizegdb.org/data_center/primer?id=171290) | [AGCACCGTTTCTCGTGAGAT](https://www.maizegdb.org/data_center/primer?id=171291) | Xu, S.-X, Liu, J. and Liu, G.S. 2004 |
| Bnlg1450 | yield/yield heterosis | 10.07 | AG(34) | [ACAGCTCTTCTTGGCATCGT](https://www.maizegdb.org/data_center/primer?id=171308) | [GACTTTGCTGGTCAGCTGGT](https://www.maizegdb.org/data_center/primer?id=171309) | Xu, S.-X, Liu, J. and Liu, G.S. 2004 |
| Bnlg1484 | yield/yield heterosis | 1.03 | AG(19) | [GTAAAAGACGACGACATTCCG](https://www.maizegdb.org/data_center/primer?id=171320) | [GACGTGCACTCCGTTTAACA](https://www.maizegdb.org/data_center/primer?id=171321) | Xu, S.-X, Liu, J. and Liu, G.S. 2004 |
| Bnlg1502 | yield/yield heterosis | 1.09 | AG(17) | [AGAGGTGGTATGATCACCTGG](https://www.maizegdb.org/data_center/primer?id=171325) | [AGGTCCTGGCACTAAGAGCA](https://www.maizegdb.org/data_center/primer?id=171324) | Xu, S.-X, Liu, J. and Liu, G.S. 2004 |
| Bnlg1606 | yield/yield heterosis | 2.08 | AG(23) | [TGTCCTTGTACCAGTGCTGC](https://www.maizegdb.org/data_center/primer?id=171374) | [GCTGTTCAGGATCTTCTGCC](https://www.maizegdb.org/data_center/primer?id=171375) | Xu, S.-X, Liu, J. and Liu, G.S. 2004 |
| Bnlg1643 | yield/yield heterosis | 1.08 | AG(24) | [ATTGACCCCGTGACCCTC](https://www.maizegdb.org/data_center/primer?id=171403) | [ACCACCGTCCACCTCCAC](https://www.maizegdb.org/data_center/primer?id=171402) | Xu, S.-X, Liu, J. and Liu, G.S. 2004 |
| bnlg1724 | yield and heterosis | 9.01 | AG(31) | [CTGACCCAGAGCATTGTGAA](https://www.maizegdb.org/data_center/primer?id=171444) | [GATGAAGAGCTTGCAGTCCC](https://www.maizegdb.org/data_center/primer?id=171445) | Fernandes et al. 2015 |
| bnlg1811 | yield and heterosis | 1.04 | AG(16) | [GTAGTAGGAACGGGCGATGA](https://www.maizegdb.org/data_center/primer?id=171491) | [ACACAAGCCGACCAAAAAAC](https://www.maizegdb.org/data_center/primer?id=171490) | Fernandes et al. 2015 |
| Bnlg2046 | yield/yield heterosis | 8.05 | AG(15) | [TTGGTGAAACGGTGAAATGA](https://www.maizegdb.org/data_center/primer?id=171568) | [CTGGTGAGCTTCACCCTCTC](https://www.maizegdb.org/data_center/primer?id=171569) | Xu, S.-X, Liu, J. and Liu, G.S. 2004 |
| bnlg2241 | yield and heterosis | 3.06 | AG(26) | [GTGCACACTCTCTTGCATCG](https://www.maizegdb.org/data_center/primer?id=171620) | [TAGTCAGCATCTGCCGTGTC](https://www.maizegdb.org/data_center/primer?id=171621) | Fernandes et al. 2015 |
| bnlg589 | yield and heterosis | 4.1 | - | [GGGTCGTTTAGGGAGGCACCTTTGGT](https://www.maizegdb.org/data_center/primer?id=114480) | [GCGACAGACAGACAGACAAGCGCATTGT](https://www.maizegdb.org/data_center/primer?id=114481) | Fernandes et al. 2015 |
| bnlg669 | yield and heterosis | 8.03 | - | [GCACGCACCAGCAGTCGGCAGT](https://www.maizegdb.org/data_center/primer?id=114636) | [CGGCCTAGTGGGCATGGAGCCT](https://www.maizegdb.org/data_center/primer?id=114637) | Fernandes et al. 2015 |
| mmc0241 | yield and heterosis | 6.05 | (TA)4N13(TG)15 | [TATATCCGTGCATTTACGTTT](https://www.maizegdb.org/data_center/primer?id=167190) | [CATCGCTTGTCTGTCGA](https://www.maizegdb.org/data_center/primer?id=167191) | Fernandes et al. 2015 |
| Phi001 | yield/yield heterosis | 1.03 | AG | [TGACGGACGTGGATCGCTTCAC](https://www.maizegdb.org/data_center/primer?id=111631) | [AGCAGGCAGCAGGTCAGCAGCG](https://www.maizegdb.org/data_center/primer?id=111632) | Xu, S.-X, Liu, J. and Liu, G.S. 2004 |
| phi014 | heterotic groups | 8.04 | GGC | [AGATGACCAGGGCCGTCAACGAC](https://www.maizegdb.org/data_center/primer?id=111639) | [CCAGCTTCACCAGCTTGCTCTTCGTG](https://www.maizegdb.org/data_center/primer?id=111640) | C. Barata and M. J. Carena,2006 |
| phi022 | heterotic groups | 9.03 | GTGC | [TGCGCACCAGCGACTGACC](https://www.maizegdb.org/data_center/primer?id=111651) | [GCGGGCGACGCTTCCAAAC](https://www.maizegdb.org/data_center/primer?id=111652) | C. Barata and M. J. Carena,2006 |
| phi026 | yield and heterosis | 4.05 | CT | [TAATTCCTCGCTCCCGGATTCAGC](https://www.maizegdb.org/data_center/primer?id=111657) | [GTGCATGAGGGAGCAGCAGGTAGTG](https://www.maizegdb.org/data_center/primer?id=111658) | Fernandes et al. 2015 |
| phi032 | heterotic groups | 9.04 | AAAG | [CTCCAGCAAGTGATGCGTGAC](https://www.maizegdb.org/data_center/primer?id=111667) | [GACACCCGGATCAATGATGGAAC](https://www.maizegdb.org/data_center/primer?id=111668) | C. Barata and M. J. Carena,2006 |
| Phi046 | yield/yield heterosis | 3.08 | ACGC | [ATCTCGCGAACGTGTGCAGATTCT](https://www.maizegdb.org/data_center/primer?id=130829) | [TCGATCTTTCCCGGAACTCTGAC](https://www.maizegdb.org/data_center/primer?id=130830) | Xu, S.-X, Liu, J. and Liu, G.S. 2004 |
| phi053 | heterotic groups | 3.05 | ATAC | [AACCCAACGTACTCCGGCAG](https://www.maizegdb.org/data_center/primer?id=130844) | [CTGCCTCTCAGATTCAGAGATTGAC](https://www.maizegdb.org/data_center/primer?id=130843) | C. Barata and M. J. Carena,2006 |
| phi061 | yield and heterosis | 9.03 | TTCT-GTAT | [GACGTAAGCCTAGCTCTGCCAT](https://www.maizegdb.org/data_center/primer?id=111687) | [AAACAAGAACGGCGGTGCTGATTC](https://www.maizegdb.org/data_center/primer?id=111688) | Fernandes et al. 2015 |
| phi072 | heterotic groups | 4 | AAAC | [ACCGTGCATGATTAATTTCTCCAGCCTT](https://www.maizegdb.org/data_center/primer?id=111701) | [GACAGCGCGCAAATGGATTGAACT](https://www.maizegdb.org/data_center/primer?id=111702) | C. Barata and M. J. Carena,2006 |
| phi093 | heterotic groups | 4.08 | AGCT | [AGTGCGTCAGCTTCATCGCCTACAAG](https://www.maizegdb.org/data_center/primer?id=111731) | [AGGCCATGCATGCTTGCAACAATGGATACA](https://www.maizegdb.org/data_center/primer?id=111732) | C. Barata and M. J. Carena,2006 |
| phi114 | heterotic groups | 7.03 | GCCT | [CCGAGACCGTCAAGACCATCAA](https://www.maizegdb.org/data_center/primer?id=111745) | [AGCTCCAAACGATTCTGAACTCGC](https://www.maizegdb.org/data_center/primer?id=111746) | C. Barata and M. J. Carena,2006 |
| phi123 | heterotic groups | 6.07 | AAAG | [GGAGACGAGGTGCTACTTCTTCAA](https://www.maizegdb.org/data_center/primer?id=130891) | [TGTGGCTGAGGCTAGGAATCTC](https://www.maizegdb.org/data_center/primer?id=130892) | C. Barata and M. J. Carena,2006 |
| Phi126 | yield/yield heterosis | 6 | AG | [TCCTGCTTATTGCTTTCGTCAT](https://www.maizegdb.org/data_center/primer?id=130895) | [GAGCTTGCATATTTCTTGTGGACA](https://www.maizegdb.org/data_center/primer?id=130896) | Xu, S.-X, Liu, J. and Liu, G.S. 2004 |
| phi127 | heterotic groups | 2.08 | AGAC | [ATATGCATTGCCTGGAACTGGAAGGA](https://www.maizegdb.org/data_center/primer?id=130897) | [AATTCAAACACGCCTCCCGAGTGT](https://www.maizegdb.org/data_center/primer?id=130898) | C. Barata and M. J. Carena,2006 |
| phi129 | heterotic groups | 6.05 | ATAC | [TCCAGGATGGGTGTCTCATAAAACTC](https://www.maizegdb.org/data_center/primer?id=130902) | [GTCGCCATACAAGCAGAAGTCCA](https://www.maizegdb.org/data_center/primer?id=130901) | C. Barata and M. J. Carena,2006 |
| phi328175 | heterotic groups | 7.04 | AGGTG | [GGGAAGTGCTCCTTGCAG](https://www.maizegdb.org/data_center/primer?id=256151) | [CGGTAGGTGAACGCGGTA](https://www.maizegdb.org/data_center/primer?id=256152) | C. Barata and M. J. Carena,2006 |
| phi96100 | heterotic groups | 2.01 | ACCT | [AGGAGGACCCCAACTCCTG](https://www.maizegdb.org/data_center/primer?id=256220) | [TTGCACGAGCCATCGTAT](https://www.maizegdb.org/data_center/primer?id=256221) | C. Barata and M. J. Carena,2006 |
| phi96342 | heterotic groups | 10.02 | ATCC | [GTAATCCCACGTCCTATCAGCC](https://www.maizegdb.org/data_center/primer?id=256223) | [TCCAACTTGAACGAACTCCTC](https://www.maizegdb.org/data_center/primer?id=256224) | C. Barata and M. J. Carena,2006 |
| Umc1014 | yield/yield heterosis | 6.04 | (GA)12 | [GAAAGTCGATCGAGAGACCCTG](https://www.maizegdb.org/data_center/primer?id=167263) | [CCCTCTCTTCACCCCTTCCTT](https://www.maizegdb.org/data_center/primer?id=167264) | Xu, S.-X, Liu, J. and Liu, G.S. 2004 |
| umc1015 | yield and heterosis | 7.03 | (GA)45 | [CAGACACAAGCAGCAAAGCAAG](https://www.maizegdb.org/data_center/primer?id=167265) | [TCCGACTCCAAGAAGAGGAGAA](https://www.maizegdb.org/data_center/primer?id=167266) | Fernandes et al. 2015 |
| umc1019 | yield and heterosis | 5.06 | (CT)17 | [CCAGCCATGTCTTCTCGTTCTT](https://www.maizegdb.org/data_center/primer?id=167273) | [AAACAAAGCACCATCAATTCGG](https://www.maizegdb.org/data_center/primer?id=167274) | Fernandes et al. 2015 |
| Umc1031 | yield/yield heterosis | 4.05 | (CT)6AT(CT)9 | [TTGGGTTCATACCTCCTAGGAACA](https://www.maizegdb.org/data_center/primer?id=174663) | [ACGTGGACAACCAGTCTATCAACA](https://www.maizegdb.org/data_center/primer?id=174664) | Xu, S.-X, Liu, J. and Liu, G.S. 2004 |
| umc1033 | yield and heterosis | 9.02 | (GA)25 | [CTTCTTCGTAAAGGCATTTTGTGC](https://www.maizegdb.org/data_center/primer?id=174707) | [GTGCGGGATTCCTTAGTTTGC](https://www.maizegdb.org/data_center/primer?id=174708) | Fernandes et al. 2015 |
| umc1035 | yield and heterosis | 1.06 | (CT)19 | [CTGGCATGATCACGCTATGTATG](https://www.maizegdb.org/data_center/primer?id=174751) | [TAACATCAGCAGGTTTGCTCATTC](https://www.maizegdb.org/data_center/primer?id=174752) | Fernandes et al. 2015 |
| umc1071 | yield and heterosis | 1.01 | (TACGA)5 | [AGGAAGACACGAGAGACACCGTAG](https://www.maizegdb.org/data_center/primer?id=193982) | [GTGGTTGTCGAGTTCGTCGTATT](https://www.maizegdb.org/data_center/primer?id=193983) | Fernandes et al. 2015 |
| umc1359 | yield and heterosis | 7.04 | (TC)12 | [GCAGAGCCAGAATTCGACCTT](https://www.maizegdb.org/data_center/primer?id=248676) | [CATCGTCATCATTCGAGCAGAG](https://www.maizegdb.org/data_center/primer?id=248677) | Fernandes et al. 2015 |
| umc1653 | yield and heterosis | 6.07 | (GAAA)24 | [GAGACATGGCAGACTCACTGACA](https://www.maizegdb.org/data_center/primer?id=291292) | [GCCGCCCACGTACATCTATC](https://www.maizegdb.org/data_center/primer?id=291293) | Fernandes et al. 2015 |
| umc1786 | yield and heterosis | 8.01 | (TC)7 | [CATTTTTCGCATTTAGGAAATCCA](https://www.maizegdb.org/data_center/primer?id=292706) | [ACCGTGACTTCCTCCTCATAACTG](https://www.maizegdb.org/data_center/primer?id=292705) | Fernandes et al. 2015 |

Supplementary table-3: Descriptive statistics for yield component traits of inbred lines

| Particular | Cob Length (cm) | Cob Girth (mm) | Kernel Row Number | Kernel Per Row | Grain yield (kg/ha) |
| --- | --- | --- | --- | --- | --- |
| Mean | 12.83 | 35.47 | 14.00 | 21.49 | 2365.36 |
| Minimum | 8.75 | 26.25 | 10.00 | 12.00 | 1087.99 |
| Maximum | 16.05 | 41.35 | 22.00 | 31.00 | 3113.20 |
| Standard Deviation | 2.13 | 4.87 | 2.47 | 5.01 | 605.52 |

Supplementary table 4: Polymorphic Information Content (PIC) value of markers

| Sl.No. | Marker | PIC value | Sl.No | Marker | PIC value |
| --- | --- | --- | --- | --- | --- |
| 1 | umc1359 | 0.62 | 26 | Bnlg1064 | 0.94 |
| 2 | mmc0241 | 0.79 | 27 | Bnlg1108 | 0.94 |
| 3 | Bnlg1138 | 0.85 | 28 | phi014 | 0.94 |
| 4 | Bnlg1502 | 0.85 | 29 | umc1071 | 0.94 |
| 5 | Phi046 | 0.85 | 30 | umc1653 | 0.94 |
| 6 | phi022 | 0.85 | 31 | Bnlg1297 | 0.95 |
| 7 | umc1786 | 0.85 | 332 | phi026 | 0.95 |
| 8 | bnlg669 | 0.85 | 33 | umc1015 | 0.95 |
| 9 | bnlg1724 | 0.85 | 34 | bnlg1811 | 0.96 |
| 10 | phi127 | 0.85 | 35 | Bnlg1175 | 0.96 |
| 11 | phi093 | 0.87 | 36 | phi072 | 0.96 |
| 12 | phi328175 | 0.87 | 37 | Bnlg2046 | 0.96 |
| 13 | phi96100 | 0.87 | 38 | Bnlg1074 | 0.96 |
| 14 | bnlg589 | 0.89 | 39 | bnlg2241 | 0.97 |
| 15 | bnlg1006 | 0.89 | 40 | phi114 | 0.97 |
| 16 | Phi126 | 0.89 | 41 | phi032 | 0.97 |
| 17 | bnlg1031 | 0.89 | 42 | phi053 | 0.97 |
| 18 | Bnlg1484 | 0.91 | 43 | umc1019 | 0.97 |
| 19 | Umc1014 | 0.91 | 44 | umc1035 | 0.98 |
| 20 | phi123 | 0.91 | 45 | Phi001 | 0.98 |
| 21 | Bnlg1429 | 0.92 | 46 | Bnlg1606 | 0.98 |
| 22 | phi061 | 0.92 | 47 | phi129 | 0.99 |
| 23 | Bnlg1450 | 0.92 | 48 | Bnlg1643 | 0.99 |
| 24 | phi96342 | 0.93 | 49 | umc1033 | 0.99 |
| 25 | Bnlg1178 | 0.93 | 50 | bnlg1045 | 0.99 |

Supplementary table 5: Grouping of inbred lines and their cluster mean for yield and yield component traits

| Clusters and Genotypes | | Cluster Mean | | | | |
| --- | --- | --- | --- | --- | --- | --- |
|  | Cob  Length (cm) | | Cob Girth (mm) | Kernel Row Number | Kernel Per Row | Grain yield (kg/ha) |
| *Cluster I*: PML 44, PML 93, PML 103, PML 111, PML 112, PML 115, DML 1913 and DML 1336 | | 13.55 | 34.92 | 14.00 | 22.01 | 2196.39 |
| *Cluster II*: PML 45, PML 102, PML 109, PML 110, PML 113, PML 114, PML 116 | | 11.45 | 36.57 | 14.29 | 19.36 | 2464.06 |
| *Cluster III*: PML 46 | | 16.05 | 31.50 | 14.00 | 30.38 | 3026.25 |
| Population Mean | | 12.79 | 35.43 | 14.13 | 21.37 | 2365.36 |
| Standard Deviation | | 2.11 | 4.85 | 2.36 | 4.91 | 605.52 |

Supplementary table 6: Yield and yield component traits for good performing hybrids

| Hybrids | Pedigree | Cob length (cm) | Cob girth (cm) | Kernel row number | Kernel per row | Grain yield (t/ha) |
| --- | --- | --- | --- | --- | --- | --- |
| AH4323 | PML116×PML93 | 18.33 | 15.53 | 16.27 | 35.73 | 12.13 |
| AH4304 | PML110×PML46 | 17.79 | 15.14 | 14.40 | 33.73 | 10.93 |
| AH4305 | PML111×PML46 | 17.73 | 14.73 | 14.27 | 32.73 | 10.93 |
| AH4316 | PML109×PML93 | 17.05 | 14.40 | 14.00 | 31.53 | 10.83 |
| AH4334 | PML114×PML102 | 16.60 | 14.23 | 12.80 | 29.13 | 10.46 |
| Bio9544 | CHECK | 17.22 | 14.07 | 13.73 | 31.40 | 7.13 |
| **CD @ 5%** | | **NS** | **NS** | **2.08** | **NS** | **2.27** |


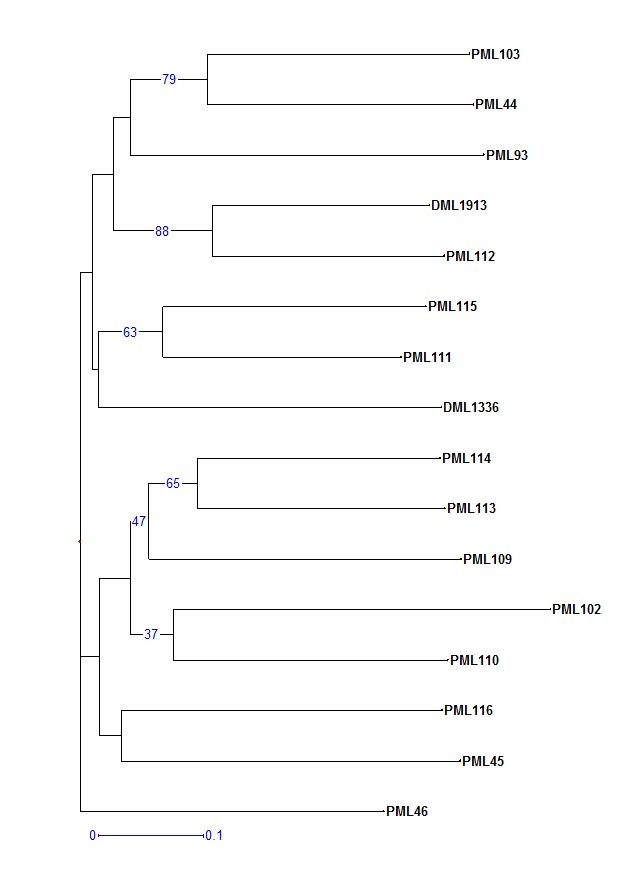


Supplementary figure 1: Dendrogram generated from an un-weighted pair group method (UPGMA) based on the molecular diversity using 50 SSR markers
